# Serological Protection Rates against TBEV Infection in Blood Donors from a Highly Endemic Region in Southern Germany

**DOI:** 10.3390/vaccines11030522

**Published:** 2023-02-23

**Authors:** Gerhard Dobler, Kathrin Euringer, Klaus Kaier, Johannes P. Borde

**Affiliations:** 1 Bundeswehr Institute of Microbiology, Neuherbergstraße 11, 80937 München, Germany; 2 Parasitology Unit, University of Hohenheim, Emil-Wolff-Straße 34, 70599 Stuttgart, Germany; 3 Division of Infectious Diseases, Department of Medicine II, University of Freiburg Medical Center and Faculty of Medicine, 79098 Freiburg, Germany; 4 Institute of Medical Biometry and Statistics, Faculty of Medicine and Medical Center, University of Freiburg, Stefan-Meier-Straße 26, 79098 Freiburg, Germany; 5 Gesundheitszentrum Oberkirch, Am Marktplatz 8, 77704 Oberkirch, Germany

**Keywords:** TBEV, TBE, Germany, epidemiology, vaccines, protection, NS1

## Abstract

Background: Tick-borne encephalitis (TBE) is the most significant tick-borne disease in Europe and Asia, with more than 10,000 cases per year worldwide. A surge of reported TBE cases can be observed despite the availability of highly efficient vaccines. There is little known about the serological immune protection rate of the population in Germany. The seroprotection rate is defined as the presence of neutralizing antibodies. In contrast, the vaccination rate, as defined by public health agencies, may differ from the true protection rate in a population. Materials and Methods: 2220 blood samples from inhabitants of the county Ortenaukreis in the Federal State of Baden-Württemberg in Germany were included in the study. These were tested for anti-TBEV IgG antibodies by an anti-TBEV-IgG-ELISA. Subsequently, all TBEV-IgG positive samples were confirmed for neutralizing antibodies in the micro serum neutralization assay. Results: From the overall 2220 samples, 2104 were included in the comparison because of the selection of specific age groups (ages 20–69). In our sample size, we found an average serological protection rate (presence of neutralizing antibodies) of 57% (518/908) for the female blood donors and of 52% (632/1196) for the male blood donors. Discussion: In this study, we present new findings on a highly endemic region in southern Germany. Additionally, we present current data regarding the serological TBEV protection rates in the Ortenaukreis in southern Germany and compare these with a dataset published by the RKI, which is based on vaccination reports of the primary care providers and health care insurers, and with a self-reporting study conducted by a vaccine manufacturer. Our results significantly exceed the official numbers of average active vaccination status by 23.2% for females and by 21% for males. This might indicate an even longer persistence of TBE-vaccination-induced antibody titers than previously assumed.

## 1. Introduction

Tick-borne encephalitis (TBE) is an emerging tick-borne disease in Europe and Asia with thousands of notified cases each year [1]. Its etiological agent is the TBE-Virus (TBEV), a single-stranded RNA virus of positive polarity (+ ss RNA). It belongs to the genus *Flavivirus* in the family *Flaviviridae* [2]. To date there are three confirmed virus subtypes, which include the European, Siberian and Far-Eastern subtypes [3]. Two other subtypes (Bakalian and Himalayan) have been proposed recently [4,5]. In Germany and Western Europe, so far only the European subtype has been detected to circulate. TBEV cycles in natural foci, between small mammals and ticks. It is typically transmitted to humans via a tick bite from *Ixodes ricinus*. In some cases, the milk of infected ungulates has caused alimentary TBEV cases in humans [6]. TBEV infection often causes sub-clinical TBE courses which remain unnoticed, or non-specific symptoms where patients may also suffer from febrile, flu-like symptoms. In up to 30% of the symptomatic cases, a biphasic course with affection of the central nervous system (CNS) can occur [7], which may cause, in more than 40% of the cases, long-term or permanent neurological sequelae [8]. The case fatality rate for an infection with the European Subtype is ~1%, calculated on the basis of the notified TBEV infections [1].

Recently published findings from a high-endemic district, the Ortenaukreis in Baden-Wuerttemberg, southern Germany, point towards a surge of TBEV antibody seroprevalence despite the broad availability of highly efficient TBEV vaccines and respective recommendations [9]. Data on the vaccination rate in the German population are difficult to obtain. There are only few sources available for the estimation of the vaccination rates against TBEV in Germany. Annually, the vaccination certificates of German schools’ starting pupils, (RKI, Epidemiological Bulletin 48/2022), are checked for vaccination against TBEV in addition to the generally recommended vaccines. In the most recent published control study (RKI, Epidemiological Bulletin 48/2022), the vaccination coverage in the federal state of Baden-Württemberg was on average 24.9% with wide ranges in the different districts. However, this rate includes only pupils with a complete basic vaccination status and a regular booster. The second source is statistics retrieved from German health insurers (RKI, Epidemiological Bulletin 47/2020). These data list exclusively persons with an active immunization status, based on the published national recommendations. However, after two vaccinations a protection rate of >80% may be achieved. On completing the basic vaccination course, the protection rate may last substantially longer than the proposed period of three (>50/60 years of age according to vaccine product) or five years (<50/60 years of age according to vaccine product), respectively, when a booster vaccination is officially recommended [10,11]. A third source is the study on vaccine coverage based on self-reporting conducted by the vaccine manufacturers. In one of these recent studies, a vaccination coverage of 27% was found for Germany with a range from 5 to 40% for the particular federal states [12]. However, this self-disclosure regarding the vaccination status by non-medical persons may be mistake-prone. In conclusion, all these surveys may miscalculate the true protection rates in the German population.

The seroprotection rate is defined as the presence of neutralizing antibodies, whereas an active vaccination status is based on the manufacturers’ recommendations for regular basic vaccination schedules and booster intervals. Thus, the protection rate is independent from defined booster intervals or schedules. In Germany, there are two approved TBEV vaccines available (Encepur^®^, Bavarian Nordic; FSME-IMMUN^®^, Pfizer). Moreover, in Germany TBEV vaccination is recommended for all people with activities in nature in the defined TBE risk districts (RKI, Epidemiologisches Bulletin 9/2022), which are mainly located in the southern federal states of Bavaria and Baden-Wuerttemberg and the northern neighboring federal states of Saxony, Thuringia, Rhineland-Palatinate and Hesse. In addition, immunological protection induced by infection may account for the overall protection rate in the population, especially in high-endemic risk areas. Our data show that >10% of anti-TBE-IgG-positive blood donors in the Ortenaukreis show anti-TBE-NS1-IgG, which indicates a past TBE infection [9]. A differentiation between infection-induced and vaccine-induced antibodies has not been possible until recently, when an assay using the detection of anti-TBEV-NS1-IgG antibodies was introduced [13,14].

To date, no systematic studies investigating the current TBEV protection status, including vaccine-induced and infection-induced protection in the general population of risk in Germany, are available. This is due to the methodological difficulties of differentiating cross-reacting flavivirus antibodies using routine assays (ELISA). For the differentiation of these antibodies the neutralization test has to be used, which is labor-intensive, and the work with viable TBEV needs BSL-3 conditions. 

In our study, we present a follow-up analysis of the recently conducted seroprevalence study in the highly endemic district Ortenaukreis, Baden-Wuerttemberg in south Germany [9]. Aside from the analysis of protection rates, we compare data from earlier studies on TBEV protection rate in the population to our findings. Additionally, neutralizing antibody seroprevalence was correlated with different age groups and gender. The results allowed us to draw conclusions about the link between antibody quantity, confirmed by enzyme-linked-immunosorbent-assay (ELISA), and antibody neutralizing capacity, proven by micro-serum neutralization assay (mSNA), as well. 

## 2. Materials and Methods

### 2.1. Blood Samples

All methods and materials, notably the acquisition of blood samples, were previously explained in detail [9]. In brief, 2220 samples from blood donors in the Ortenaukreis, Baden-Wuerttemberg, Germany, were collected from May to December 2021. The donors’ ages ranged from 18 to 72 years. As every donor gave written consent to make the anonymized test samples available for scientific purposes, no ethics approval was required. 

### 2.2. Active Vaccination Status

Results on the vaccination coverage status were obtained from three different sources: the first source, the most recently published analysis of the students vaccination status, published by the RKI (RKI, Epidemiological Bulletin 48/2022); the second source, from the dataset on vaccination coverage as provided by the RKI and health insurers (RKI, Epidemiological Bulletin 47/2020); and the third source, from a survey based on self-reporting as provided by the vaccine manufacturers [11]. Based on the RKI and health insurers dataset (RKI, Epidemiological Bulletin 47/2020), the RKI provided an edited set of data representing the region/district of our interest—the Ortenaukreis, where the blood samples were collected [15].

### 2.3. Serological Methods

All 2220 samples were screened for TBEV-IgG antibodies with a conventional anti-TBEV-IgG-ELISA (Euroimmun, Lübeck, Germany). Positive and equivocal samples were subsequently tested in the recently validated anti-TBEV-NS1-IgG-ELISA [12]. With this assay, infection-induced antibodies were differentiated from vaccine-induced antibodies. All samples that reacted for TBEV-IgG antibodies, but not in the anti-TBE-NS1-IgG assay, were tested by mSNA in order to detect neutralizing antibodies and, therefore, confirm seroprotection [16]. For neutralization, 50 to 100 tissue culture infected doses of TBEV strain Neudoerfl were added to each serum in dilution 1:10 and 1:20. Samples with a titer of ≥20 in the mSNA were counted as vaccinees. As, to our experience, sera with specific antibodies to other flaviviruses (e.g., dengue virus, yellow fever virus, Japanese encephalitis virus, West Nile virus) may cross-react with low titers (1:10) in the mSNA, sera with titers of 1:10 were titrated by indirect immune fluorescence against different flaviviruses (Flavivirus Mosaic, Euroimmun, Luebeck, Germany) according to the manufacturer’s instructions to exclude IgG antibodies against other cross-reacting flaviviruses.

For the titration in mSNA, sera were diluted from 1:10 to 1:1280 in twofold dilutions and tested in duplicate. The highest serum dilution which neutralized the virus was taken as the neutralizing titer.

### 2.4. Data Analysis

IBM SPSS version 28.0.0.0 was used to analyze the datasets. Descriptive statistics were calculated, and furthermore a linear regression model was applied to investigate the link between ODs and age, as well as SNA titers and age. Displayed are R^2^ and ANOVA test results proving or disproving the putative null-hypothesis. 

## 3. Results

TBEV-IgG protection rates

**Figure 1:**
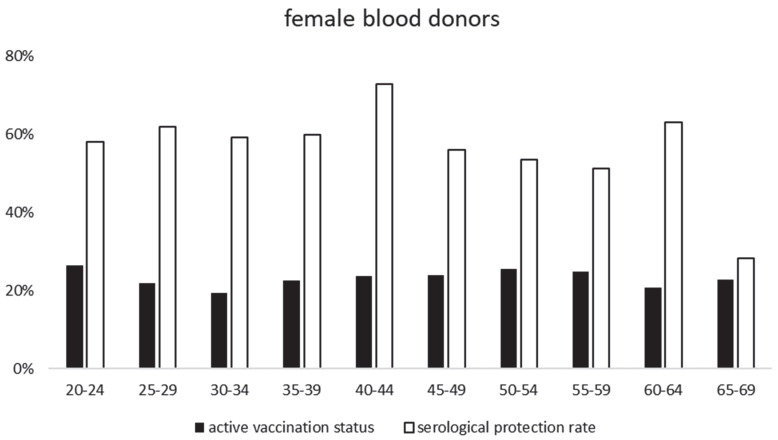
shows the comparison of active vaccination status and serological protection rate found in our study—female blood donors.

**Figure 2:**
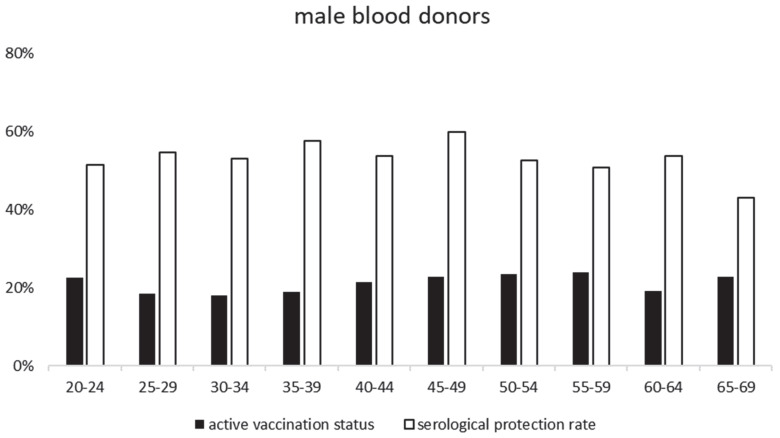
shows the comparison of active vaccination status and serological protection rate found in our study—male blood donors.

TBEV-IgG antibodies in the screening ELISA were detected in 57% (1257/2220) of the samples. Almost all (1244/1257) of these showed protective TBEV-IgG antibodies in the mSNA—see Table 1 for an overview. From these 1244 samples, which have been confirmed by mSNA as anti-TBEV specific, 126 sera were matched to age distribution and then were titrated in detail for further statistical analysis. Compared to the published data on the vaccination status of the RKI and health insurers, we saw a higher TBEV neutralizing seroprotection rate across all age groups and both genders. In detail, the survey data showed for males an average vaccination status of 21% and for females 23%, whereas our study found average TBEV-IgG protection rates for males of 52% and for females 57% (see Figure 1 and Figure 2, and Tables S2 and S4). The protection rates were almost constant throughout all age groups; however, there was a small decline seen in the age group of 65–69 years. This reduction in the protection seems mainly to affect the female population of our study.

Optical density (OD) values, mSNA titers and age

For the linear regression analysis of OD values and mSNA titers, we used a subset of our data including 126 blood donors’ samples, with detailed mSNA titers. There is a significant correlation between the OD values and mSNA titers: the R^2^ is 0.24, e.g., 24% of the variance of individual mSNA titers could be explained by our applied linear regression model. For this effect, the ANOVA test shows a *p* < 0.001. Using this subset (*n* = 126) of blood donors for the comparison of mSNA titers and age, there was no significant correlation in the ANOVA test.

Optical density (OD) values and age

**Figure 3:**
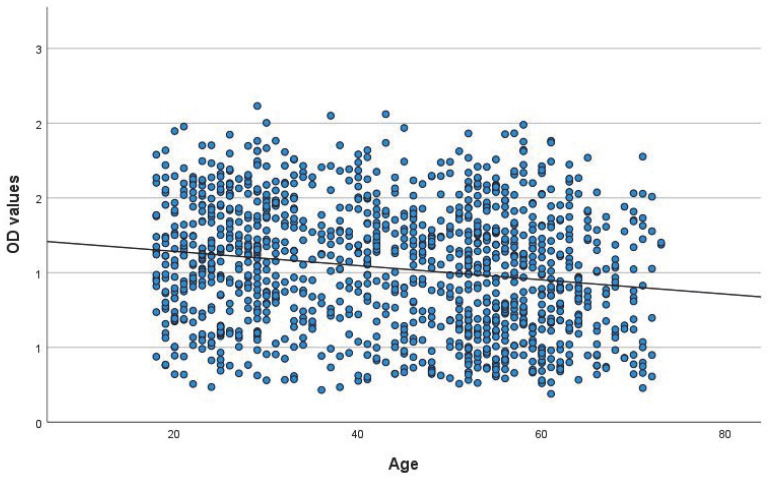
A linear regression model (line) was applied to analyse the relationship between OD values (scatter diagram) and age. There is a declining trend in OD values with increasing age.

We applied a linear regression model to investigate the relationship between age and OD values. The scatter diagram is shown in Figure 3. There is a declining trend in OD values with increasing age. The R^2^ is 0.03, e.g., only 3% of the variance of individual OD values could be explained by our applied linear regression model. For this effect, the ANOVA test shows a *p* < 0.001. 

## 4. Discussion

The German public health authorities recommend the TBEV vaccination for tick-exposed persons in so called “high risk regions (districts)” in Germany, as defined by the RKI (RKI, Epidemiologisches Bulletin, 9/2022). The basic immunization schedule for TBEV consists of three vaccine shots. The first booster is recommended after three years; further booster vaccinations are recommended after three or, respectively, five years, depending on the age group and the vaccine product. Despite these recommendations the number of human TBE cases has shown an increasing trend during the last years [17]. Therefore, the protection rate does not seem high enough in the German population at risk to decrease the number of human infections. In contrast to Austria, for Germany there are no exact data available on the TBEV protection rates. In Austria, since the introduction of the TBEV vaccine, the high field protection rates of >80% have decreased the numbers of notified human TBE cases by 80% [18,19]. 

For Germany, as well as for many other countries, only surrogate data on the assumed field protection rate against TBEV infections are available. In regular controls of the vaccination certificates of pupils in the Federal State of Baden-Württemberg, the RKI found a complete and up-to-date TBE vaccination course of about 27%. In the district Ortenaukreis the vaccination rate was reported to be below 30% (RKI, Epidemiological Bull., 48/2022). In a cross-sectional study on self-reported vaccine coverage, for the Federal State of Baden-Württemberg a vaccination rate of 37% was shown [12]. This seems to be in contrast to the results of Erber et al., where the vaccination rate in the age group <17 years was almost 50% while in the following age groups the vaccination rate decreased dramatically to only 20 to 30% [12]. These data may also show that the self-reporting of vaccination coverage may be misleading and the findings may be interpreted with caution. 

All reported data on vaccination rates in the Federal State of Baden-Württemberg stand in contrast to our results. In our study sample, the mean TBEV-IgG protection rate of all age groups is about 55%. Overall, female blood donors have slightly higher rates than the males, which is also in line with the findings from the RKI survey, where the difference in vaccination status was 21% for the male participants and 23% for female participants. 

The main reason for the discrepancy is that the field protection rate (i.e., detection of neutralizing antibodies) might be different from the vaccination rate or active vaccination status (having the full vaccination course and boosters in time). It is well known that neutralizing antibodies may persist longer than the recommended booster intervals of five years or three years depending on age [20]. Schosser et al. found, in this context, sustained protective antibody titers in the group with three or more vaccine doses, even after formally prolonged time periods [20]. Titers were significantly higher in younger than in older vaccinees. 

In the study of Erber et al., the second vaccination dose in the primary vaccination course (about four weeks after the first dose) was provided to about 80%, while the third dose (provided after 6 to 12 months) was applied only in about 60% of cases and boosters in only <30% of the participants [12]. These data fit well with the reported vaccination coverage, but may not reflect, therefore, the true field protection rate. We believe that our data are closer to the biological reality in the population than the surrogate active vaccination or vaccination coverage data. 

The OD of the anti-TBEV-IgG ELISA (Euroimmun) is a parameter resembling the quantity of anti-TBEV antibodies in a serum sample. As we have confirmed previously, 98% (1223/1244) of the samples that tested positive in the anti-TBEV-IgG-ELISA could ultimately be confirmed for TBEV specific neutralizing antibodies via mSNA [9]. In conclusion, the anti-TBEV-IgG ELISA showed a very high sensitivity and specificity in our study. The presence of anti-TBEV-IgG antibodies in serum samples is linked to the presence of a sufficient neutralizing capacity for 98% of our samples, and there is a significant correlation between OD and mSNA titers. Therefore, the anti-TBEV-IgG ELISA might be a suitable epidemiological tool to indicate serological protection for TBEV and confirm vaccine protection to a high degree in regions where only TBEV is circulating, while in individual patients with a potential medical history of other flavivirus infections or vaccinations other assays for screening and confirmation of specific anti-TBE antibodies may be used. Our study, therefore, could show that this assay may be useful for epidemiological studies, although its use for the individual patient may be limited.

Assuming a much higher field protection rate as reported by surrogate data may also pose a different assessment of risk of infection to the population. Even if assuming that only about a quarter of the population is protected and about 75% are at risk, the true incidence might be much higher when about twice as many people within the population exhibit seroprotection against TBE infection. Assuming a seroprotection rate of >50%, the true incidence rate will double. Therefore, the risk of infection and human disease might be highly underestimated in regions with low vaccination protection (as calculated by public health services), but higher seroprotection rates. 

Our study has several limitations. Our sample sizes vary for each age group and may be too small to be representative of the general population in the Ortenaukreis (Table S4). Furthermore, it must also be kept in mind that our samples come from blood donors, who usually belong to a very altruistic and health-conscious group that is in good physical shape, although no data exist to compare the TBEV vaccination rates in blood donors and the general population. Nevertheless, there might be some bias towards a higher vaccination and protection rate than exists in the non-blood donor population. The problem of persisting neutralizing antibodies over the period of booster recommendation has been discussed. At the moment, it is not clear if higher anti-TBEV neutralizing antibody titers also provide a longer lasting protection. Furthermore, data on the dynamics of the antibody decrease over time beyond the recommended booster intervals are not available. However, antibodies induced by an incomplete or irregular vaccination scheme may be sufficient for protection, at least in part, to those vaccinated. The assumption that it might not be necessary to follow the rather strict vaccination schedule for TBEV vaccination, especially for TBEV vaccine boosters, in order to be protected against the disease underscores the quality/effectiveness of the two licensed and available TBEV vaccine products. As mentioned above, data show that >10% of anti-TBEV-IgG-positive blood donors in the Ortenaukreis show anti-TBEV-NS1-IgG [9]. We did not subtract the proportion of anti-TBEV-NS1-IgG positive samples from the total amount of anti-TBEV-IgG-positive blood donors. To date, it is not evident if TBEV vaccines induce a sterile immunity (or not) regarding anti-TBEV-NS1-IgG seroconversion. Moreover, there is the possibility that blood donors began their basic vaccination schedule after an (unknown/asymptomatic) TBEV infection, which would result in an anti-TBEV-IgG-positive and anti-TBEV-NS1-IgG positive status as well. This lack of clarity must be kept in mind when interpreting our dataset and deriving epidemiological conclusions.

Taken together, our study shows that serological studies on the prevalence rate of neutralizing antibodies provide more detailed data on the field protection rate than the available studies using different data sources and methods. These studies may underestimate the field protection rate in an area with low to moderate vaccination coverage. In our study, the ELISA assay for the detection of anti-TBE-IgG antibodies proved to be a good indicator for prevalence studies. Only a small percentage of positive reactions proved to be non-specific due to other flavivirus infections or vaccinations. This might, however, be due to the situation that, at the study site, only one single flavivirus is circulating. In this specific epidemiological setting with only one circulating flavivirus, the ELISA might be a suitable indicator for use in epidemiological studies. For an individual diagnosis, the potential cross reactions with other flaviviruses, e.g., after vaccinations, must be considered. The ELISA IgG ODs correlates with the neutralization titers, although it is, to date, not clear if higher titers lead to a longer duration or broader range of protection. The underestimation of the field protection rate may also lead to an underestimation of the infection risk and incidence of TBE in the non-immune population.

## 5. Conclusions

In Germany, data on the protection rate against TBEV in the population are not available. Surrogate data are obtained by control of vaccination certificates of first grade students, by billing documents of health insurances or by self-reporting studies. In the current study, the seroprotection rate in blood donors has been studied as measured in neutralization assay. The higher seroprotection rate found in contrast to the vaccination rates found in mentioned sources may be due to the specific population in our study, but also due to different assumptions made for the protection duration after vaccination and boosting. Our data also imply much higher TBEV infection rates in endemic risk areas. In an endemic situation with only one circulating flavivirus, the cross-reaction prone ELISA might be an efficient, cheap and easy-to-perform assay for studying the seroprotection rate in a population.

## Figures and Tables

**Table 1:** Age and sex structure of the vaccinated donors.

Age-Group (Years)	TBEV-IgG Positive	TBEV-IgG Negative	TBEV-IgG Borderline	TBEV-IgG Positive or Borderline and NS1 Negative or Borderline and:		
				SNA Titer > 20 (Vaccinees)	SNA Titer ≤ 10	No IgG-Antibodies or NS1 pos. (no mSNA Performed)
18	70% (16/23)	30% (7/23)	0% (0/23)	65% (15/23)	0% (0/23)	35% (7/23)
19–24	64% (180/281)	33% (94/281)	2% (7/281)	58% (162/281)	0% (1/281)	42% (118/281)
25–29	63% (167/266)	34% (91/266)	3% (8/266)	57% (152/266)	1% (2/266)	42% (112/266)
30–34	58% (110/191)	39% (74/191)	4% (7/191)	55% (106/191)	1% (1/191)	44% (84/191)
35–39	60% (64/106)	35% (37/106)	5% (5/106)	58% (62/106)	0% (0/106)	41% (44/106)
40–44	63% (97/154)	29% (45/154)	8% (12/154)	60% (92/154)	5% (8/154)	35% (54/154)
45–49	58% (94/162)	35% (56/162)	7% (12/162)	58% (94/162)	2% (4/162)	40% (64/162)
50–54	56% (173/311)	40% (123/311)	5% (15/311)	51% (160/311)	3% (9/311)	55% (142/311)
55–59	48% (148/308)	44% (136/308)	8% (24/308)	50% (154/308)	2% (7/308)	48% (147/308)
60–64	56% (122/217)	39% (85/217)	5% (10/217)	57% (123/217)	1% (3/217)	42% (91/217)
>65	43% (86/201)	51% (103/201)	6% (12/201)	43% (87/201)	1% (2/201)	56% (112/201)
Total	57% 1257/2220	38% 851/2220	5% 112/2220	55% 1207/2220	2% 37/2220	43% 975/2220

## Data Availability

Data are available by the authors on request.

## Supplementary Materials

The following supporting information can be downloaded at: https://www.mdpi.com/article/10.3390/vaccines11030522/s1, Figure S1: Distribution of the 2104 bloodsamples (aged 20–69) from Ortenaukreis in terms of sex and serological immune protection status; Table S1: Active vaccination status as evaluated by the RKI on the basis of primary care data; Table S2: Serological protection rate as evaluated from our study sample; Table S3: Comparison of the official vaccination rates vs. serological protection rates; Table S4: Numbers of percentages of serological protection rate in our study sample (samples confirmed serological protection/total number of samples).
