# Supplementary material for: Serological Protection Rates against TBEV Infection in Blood Donors from a Highly Endemic Region in Southern Germany
Source: Vaccines (Basel). 2023 Feb 23;11(3):522. doi: 10.3390/vaccines11030522 (PMC10056601; doi:10.3390/vaccines11030522)
Supplement: Supplementary file 1 [file vaccines-11-00522-s001.zip › vaccines-2205792-supplementary.pdf]

Supplementary Material

Table S1. Active vaccination status as evaluated by the RKI on the basis of primary care data.

Age group	Male (%)	Female (%)
20-24	22.5	26.4
25-29	18.5	21.9
30-34	18.0	19.5
35-39	18.9	22.7
40-44	21.5	23.7
45-49	22.8	24.1
50-54	23.5	25.5
55-59	23.9	24.8
60-64	19.1	20.9
65-69	22.7	22.8
Mean value	21.14	23.22

Table S2. Serological protection rate as evaluated from our study sample.

Age group.	Male (%)	Female (%)
20-24	51,5	58,16
25-29	54,7	61,9
30-34	53	59,2
35-39	57,5	60
40-44	53,75	73
45-49	59,77	56
50-54	52,6	53,6
55-59	50,8	51,2
60-64	53,7	63,2
65-69	43	28,2
Mean value	52,2	57,0

Table S3. Comparison of the official vaccination rates vs. serological protection rates.

Age group	Male (%)	Samples: confirmed serological protection/ total number of samples
20-24	51,5	50/97
25-29	54,7	81/148
30-34	53	61/115
35-39	57,5	41/71
40-44	53,75	43/80
45-49	59,77	52/87
50-54	52,6	91/173
55-59	50,8	93/183
60-64	53,7	80/149
65-69	43	40/93
In total:	52,2	632/1196

Table S4. Numbers of percentages of serological protection rate in our study sample (samples confirmed serological protection/ total number of samples).

Age group	Female (%)	Samples: confirmed serological protection/ total number of samples
20-24	58,16	89/153
25-29	61,9	73/118
30-34	59,2	45/76
35-39	60	21/35
40-44	73	54/74
45-49	56	42/75
50-54	53,6	74/138
55-59	51,2	64/125
60-64	63,2	43/68
65-69	28,2	13/46
In total:	57,0	518/908

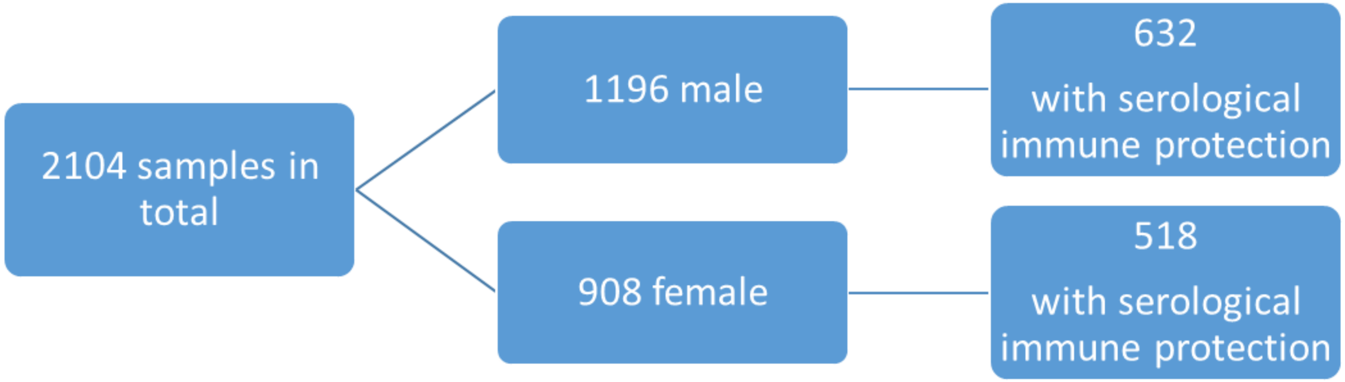

Figure S1. Distribution of the 2104 bloodsamples (aged 20-69) from Ortenaukreis in terms of sex and serological immune protection status.
